# A model to address healthcare gaps and poor research participation in Hispanic patients with Parkinson's disease

**DOI:** 10.3389/fnagi.2025.1610448

**Published:** 2025-08-12

**Authors:** M. Felice Ghilardi, Alessandro Di Rocco, Jennifer Fearday, Donna Sperlakis, Maria Lima, Nora Vanegas-Arroyave, Elisa Tatti, Marcelo Arroyave, Evelyn Stevens, Denise Hernandez, Paulina Latapi-Gonzalez, Meri-Margaret Deoudes, Christiana Evers

**Affiliations:** ^1^Department of Molecular, Cellular & Biomedical Sciences, CUNY School of Medicine, New York, NY, United States; ^2^Movement Disorders Division, Department of Neurology, Northwell Health, Donald and Barbara Zucker School of Medicine at Hofstra/Northwell, Long Island, NY, United States; ^3^Parkinson's Foundation, New York, NY, United States; ^4^Department of Neurology, Baylor College of Medicine, Houston, TX, United States; ^5^Dallas Fort Worth Community Health Worker Association, Dallas, TX, United States; ^6^Department of Neurology, Feinberg School of Medicine, Northwestern University, Chicago, IL, United States

**Keywords:** *promotores de salud*, Latino, Hispanic/Latino, healthcare improvement, Parkinson disease, research participation, community intervention, community health worker

## Abstract

Parkinson's disease (PD), a progressive neurodegenerative disorder, manifests with motor and non-motor symptoms. Despite similar incidence, in the US, disease management of Hispanic patients with PD is poorer than that of White non-Hispanics, and their participation in clinical trials does not reflect expected values (well below 5% vs. expected values around 20%). Together with other evidence, these observations are expressions of poor healthcare in Hispanic patients with PD, suggesting underutilization of proper healthcare, social and economic disadvantage, and other factors. The scant participation of Hispanic patients in clinical trials further exacerbates care inadequacy. In this paper, after reviewing evidence of healthcare gaps in Hispanic patients with PD and their low participation in research, we present a comprehensive model for reaching these patients and their caregivers, educating them on PD, and engaging them in research. This model is based on the work and experience of the Parkinson's Foundation, which over the years, has worked to increase PD awareness in Hispanic communities. This model is based on the training of *promotores de salud* (community health workers) inside Hispanic communities, the identification of needs and priorities of patients and caregivers with surveys and focus groups within Hispanic communities, and the patients' feedback about their participation in research. The development of this model could achieve sustainable partnerships with community-based organizations to promote better care and enhance participation in research of Hispanic patients. In turn, this could lead to a better quality of life for all patients with PD and their caregivers.

## 1 Introduction

Parkinson's disease (PD), the world's fastest-growing neurodegenerative disease, is a progressive condition characterized by motor and non-motor manifestations. The incidence of PD in the US appears to be similar amongst White and Hispanic/Latino peoples (for simplicity, we will use “Hispanic”; De Lau and Breteler, 2006; Aamodt et al., 2023), defined as Spanish-speaking people of Central and South American origin. Nevertheless, the best predictors for optimal care are high social and economic level, marital status, urban living, and being White (Ojukwu et al., 2021). Within the US Hispanic community, which often has incomes below the national median, management of motor and non-motor PD symptoms lags behind that of White non-Hispanic patients. In addition, the participation of Hispanic patients with PD in clinical trials is disproportionately low, with percentages varying from 1.6 to 5.4% (Schneider et al., 2009; Kurlawala et al., 2021), despite Hispanics representing at least 18% of the US population (De Lau and Breteler, 2006). This discrepancy may reflect limited healthcare access or underutilization of healthcare services, social and economic disadvantages, and cultural perception of PD manifestations (Duarte Folle et al., 2023). Unless we address this critical gap, the generalizability and validity of trial results in PD will remain limited, and Hispanic patients with PD will remain caught in a vicious cycle of inadequate care. This is rather crucial, as the Hispanic population is one of the fastest-growing demographic groups, fueled by factors such as immigration, higher birth rates, and a youthful population. A notable increase in life expectancy parallels this demographic trend, thus increasing the urgency to devise ways for greater access to care and participation in clinical trials of Hispanic patients with PD. Here, we first review the evidence of healthcare gaps in Hispanic patients with PD, studies underlying their low participation in research, and the attempts to foster their engagement in clinical trials. We then describe a possible model to address these problems based on the experience of recent initiatives by the Parkinson's Foundation.

## 2 Healthcare gaps in Hispanic patients with PD

Several analyses of available data sets have now provided evidence that Hispanic patients with PD may receive inadequate quality of health care compared to White non-Hispanic patients. As an example, a recent review of discharge data (from 2012 to 2018) of the National Inpatient Sample, Healthcare Cost and Utilization Project, Agency for Healthcare Research and Quality showed that, among many healthcare inequities, Hispanic patients with PD are less likely to be treated with deep brain stimulation than White patients (Dorritie et al., 2023), confirming earlier findings (Willis et al., 2014). Also, there are only a few clinical studies or trials focused on Hispanic patients with PD, and only in one of them, which combined two cohorts followed for a few years in two different periods in California, Hispanic patients were present in representative proportion (18%, corresponding to *N* = 138; Duarte Folle et al., 2023). That longitudinal study showed that in Hispanic patients, PD symptoms and signs appear at a younger age, progress faster in terms of the Hoehn & Yahr scale, and are, on average, more severe compared to their non-Hispanic counterparts (Duarte Folle et al., 2023). Moreover, studies indicate that in a large proportion of Hispanic patients, little or no drug adjustment occurs with the progression of the symptoms (Duarte Folle et al., 2023), even though changes in pharmacological treatment can improve life quality, prevent comorbidities and delay disability. This suboptimal therapeutical management may stem from a lack of financial resources, language and cultural obstacles, and difficulty in accessing adequate medical facilities, prescriptions, and medications. Indeed, studies have found that Hispanic patients with PD are 40% less likely to seek neurological care (Saadi et al., 2017) and in particular, to be cared for by a movement disorders specialist (Willis et al., 2011). This population also has lower access to nursing facilities than White non-Hispanic patients (Kumar et al., 2024). Altogether, these care differences may account for faster time to disability and earlier age at death in Hispanic patients with PD (Duarte Folle et al., 2023). Since the Hispanic population is the fastest growing in the U.S., as per the 2020 Census, finding strategies to improve quality and access to movement disorder care is imperative.

The impact of PD extends beyond motor symptoms. Depression is a frequent non-motor symptom of PD that may precede the appearance of motor symptoms. Notably, depression is more frequent and severe in Hispanic than in White non-Hispanic patients with PD (Duarte Folle et al., 2023; Jones et al., 2023), a finding in line with the fact that people of Hispanic origin display the highest rates of depression compared to non-Hispanic populations (Hooker et al., 2019). The higher depression rate in Hispanic PD patients can be accounted for by the lower use of antidepressant drugs (Cheng et al., 2008; Duarte Folle et al., 2023), thus pointing to underutilization of medical services of Hispanic people, another potential gap in their healthcare quality. Importantly, reduced care for depression significantly increases the risk of developing other non-motor signs of PD, such as cognitive impairment (Babulal et al., 2022). Indeed, a study following for some years a substantial number of Hispanics with PD, reported that Hispanic patients with PD had lower scores in cognitive tests (Duarte Folle et al., 2023). These gaps in healthcare quality likely have significant negative impacts on the Hispanic patient population.

In summary, Hispanic patients with PD, on average, experience lower quality of clinical care, including delayed diagnosis, poorer care, and inadequate treatment for both motor and non-motor symptoms. Limited access to proper healthcare, social and economic disadvantage, language barrier, and perception of both the disease's clinical manifestations and the healthcare environment may be the major contributors to healthcare problems in PD. These observations highlight the inadequate care of Hispanic patients with PD and the critical need to develop strategies to improve their use of the healthcare system.

## 3 Healthcare gaps in Hispanic patients with PD parallel low participation in clinical trials

According to the 2020 Census, Hispanic people living in the US were about 62 million (18.9% of the population), the second largest racial or ethnic group after White non-Hispanic people. The Hispanic group includes persons of Mexican, Puerto Rican, Central or South American, Cuban, or other Spanish culture or origin, regardless of race. Mexicans are highly represented with 61.6%, followed by Puerto Ricans (9.6%), Central Americans (9.3%), South Americans (6.4%), other Hispanics (including Spanish; 5.8%), and Cubans (3.9%). New York State, our base of work, has the fourth largest Hispanic population in the United States, with more than 2.4 million in New York City (more than 25%) and 4.8 million in the metro area. Considering areas that are part of the larger New York region up the Connecticut coastline, the number increases to 5.3 million, representing 22% of the region's population. The distribution of the Hispanic population in New York City includes, in decreasing order, Puerto Ricans and Dominicans, followed by Mexicans, Guatemalans, Salvadorians, Ecuadorians, and other origin, with no group reaching a clear majority. Importantly, according to the population estimate of the US Census of July 2022, Hispanic people represented more than 56% of the total population.

Most publications suggest that Hispanic and White non-Hispanic peoples have a similar incidence of PD (i.e., 1% of people older than 60 years; Van Den Eeden, 2003; De Lau and Breteler, 2006). Thus, based on the demographics outlined above, 20% of the participants in clinical trials for PD should be Hispanic patients. Yet, Hispanic research engagement is much lower, with most studies not defining all populations' characteristics. Indeed, a meta-analysis of 239 clinical trials in PD found that only 33 studies—for a total of almost 7,500 participants—specified all populations' characteristics (Schneider et al., 2009). Notably, only eight of the 33 studies included Hispanics/Latinos, for a total of 27 patients, with percentages varying from 1.6 to 5.4% (Schneider et al., 2009), in line with the 2.8% rate of Hispanic participants recruitment observed in the large-scale Parkinson's Progression Markers Initiative (PPMI) multisite study (Kurlawala et al., 2021). Most recently, two clinical trials, the Steady-PD III and the SURE-PD3, were not more successful in recruiting Hispanic participants despite the explicit use of direct or indirect incentives for enrolment: in both studies, only 2.9% of the participants were Hispanics (12 out of 413 and 17 out of 585, respectively; Di Luca et al., 2023). These findings show that, based on ethnic/racial population composition, the recruitment rate of Hispanics in the US is far below expectations, thus lessening the generalizability and validity of the results for persons with PD.

Why is fostering Hispanic patients' enrollment in clinical trials so difficult? While translating study and recruitment materials into Spanish overcomes the language barrier, other factors, such as different views about aging, family involvement, and perception of disease, are in play. The low involvement of Hispanic patients in clinical trials is likely linked to healthcare gaps and their possible causes, as illustrated above. Several studies have used strategies to address social barriers, such as language problems, yet with scant results. To effectively increase the involvement of Hispanic patients in clinical trials, other factors beyond language barriers need to be addressed, such as low healthcare literacy, mistrust in the healthcare system, investigators, and sponsors, lack of clinical trial awareness, poor communication; ineffective recruitment tools, and the concerns associated with disease (Adrissi and Fleisher, 2022; Vaswani et al., 2020; Picillo et al., 2015; Fleisher et al., 2014; George et al., 2014). The picture is further complicated by the patient's difficulty in accessing specialized movement disorders centers, together with a growing paucity of neurologists and movement disorders experts. Therefore, there is an urgent need to develop strategies for effective recruitment and retention of Hispanic patients with PD.

Recent studies have assessed strategies to increase and diversify recruitment for PD clinical studies. For example, Fox Insight used web-based digital marketing campaigns to increase Hispanic participation and the participation of people of lower educational and social and economic levels (Dobkin et al., 2020). It has been suggested that the continuous use of newsletters, social media, and PD-related websites—methods defined as “*passive recruitment*” tools—must be coupled with the so-called “*active recruitment*” methods that include *networking with local clinicians or other health providers, outreach during PD support groups, and partnerships with community organizations* (Picillo et al., 2015). Indeed, changing the environment of the recruitment from the clinic/hospital to more familiar surroundings (Nwabuobi et al., 2019; Fleisher et al., 2018), and directly involving the family with appropriate approaches and language settings (Damron et al., 2021), create a better foundation for building relationship, facilitating trust, and addressing most concerns. Also, engaging community stakeholders, such as those in religious centers, barbershops, beauty parlors, and community centers, can foster recruitment and retention in all communities (Jones and Wells, 2007; Levkoff and Sanchez, 2003; Mensah et al., 2018). In addition to the utilization of community opinion leaders and patient advocates during recruitment and, if possible, early in the study design (Feeney et al., 2020), community physicians, such as internists, primary care physicians, and neurologists, undoubtedly can play an important role. Thus, strategies for effective recruitment and retention of Hispanic patients with PD must be designed to involve community physicians and consider their time, resources, as well as patient concerns (Ison et al., 2024). Such strategies may include supporting research coordinators and implementing local training and educational events for physicians (Tilley et al., 2012).

Together, these studies and observations support the development of strategies that integrate clinical research within the patients' local community and milieu to increase the possibility of recruiting and retaining more Hispanic patients with PD in clinical trials and improve their access to optimal PD care.

## 4 The Parkinson's Foundation model

**Figure 1:**
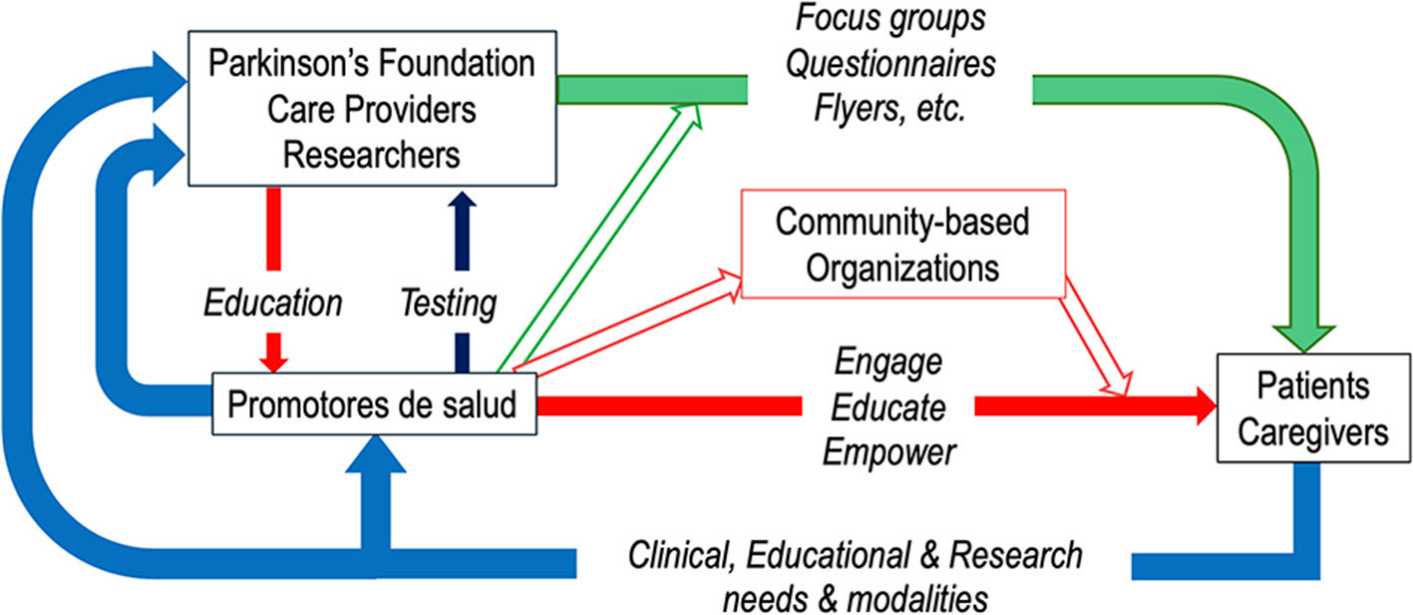
The model of the Parkinson's Foundation to address gaps in care and research participation of Hispanic patients with PD. This model is based on three lines of intervention. The first line is about the training and activities of the *promotores de salud* (in red). The promotores receive training from the Parkinson's Foundation, care providers, and researchers; provide feedback about the achieved knowledge; establish links with community-based organizations and build an infrastructure to engage, educate, and empower patients and caregivers in the Hispanic community. A second line (in green) exploits all contacts of the Parkinson's Foundation and other sources and uses different means to identify the needs and priorities of patients and caregivers. The third line (in light blue) is based on the collection of direct feedback from patients and caregivers to guide research objectives, modalities of patient participation in research, and clinical and social interventions.

People with PD who identify as Hispanic or Latino have been under-resourced not only in Spanish-language education on PD but also in Spanish-speaking providers and support services. The Parkinson's Foundation has been serving Spanish-speaking people with PD for more than 20 years, establishing a variety of programs and resources to better reach people of the Hispanic community, remove obstacles to care, open research participation, and ensure that information and resources are easy to find for all (for example, see: https://www.parkinson.org/espanol). The proposed model (see Figure 1) is based on three main activities: the training of the *promotores de salud*; the identification of needs and priorities of patients and caregivers with surveys and focus groups within the Hispanic communities, and feedback from Hispanic patients about their participation in research.

### 4.1 Training the “*promotores de salud*”

Leveraging partnerships with different local organizations, the Parkinson's Foundation has used the *promotores de salud* to engage health educators already embedded in the Hispanic community. Community health worker models have been utilized in communities across the country and internationally to effectively reach various communities in different fields of medicine (Cosgrove et al., 2014). In the Hispanic community, *promotores de salud* are recognized as lay health workers who work in Spanish-speaking communities and are community leaders, motivators, health educators, champions for health literacy, and community advocates. Like other community health worker models, the *promotores de salud* program is based on the tested concept that trusted individuals from targeted communities can be the best conduit for outreach, communication, and education (Sapienza et al., 2007).

Since early 2022, the Parkinson's Foundation has provided peer-to-peer education and continuous support on PD education and care in Texas, Arizona, and Illinois, educating and training more than 350 *promotores de salud* in collaboration with local community organizations. During training sessions conducted in Spanish (see an example in Table 1), participants acquired basic knowledge of PD, identified resources available, and established talking points enabling them to deliver PD education to their community. Faculty included researchers, clinicians, and community health workers who have successfully partnered with Hispanic communities to increase knowledge about PD and engagement in research and care. After the training, *promotores* were provided with educational materials and resources to return to their communities and build on their outreach programs. Some preliminary results (Aranda et al., 2024) demonstrated the success of this initiative: following the training, analyses of the *promotores'* questionnaires revealed an increase in PD knowledge and symptom awareness. Moreover, the number of accesses to resources and educational materials of the Parkinson's Foundation (among others: Recursos webpage, Helpline, and Parkinson.org/10 Signos) also increased. By educating trusted individuals within the Hispanic community, this training program can engage and inform about PD Hispanic patients and their families.

**Table 1:** An example of schedule and agenda “La enfermedad de Parkinson 101: cómo hablar sobre el Parkinson en su comunidad.”

**Time**	**Activity**
9:30 a.m. (30 min)	Sign in, breakfast, networking
10:00 a.m. (10 min)	Introduction *Parkinson's Foundation staff*
10:10 a.m. (60 min)	Parkinson's disease: introduction, causes, factors, symptoms, care *Movement Disorder Specialist*
11:10 a.m. (10 min)	BREAK
11:20 a.m. (30 min)	How to manage PD and what resources are available? *Parkinson's Foundation staff*
11:50 a.m. (20 min)	Q&A
12:10 a.m. (30 min)	Activity 3-resources *Parkinson's Foundation staff*
12:40 a.m. (20 min)	Report out
1:00 p.m. (5 min)	Conclusions *Parkinson's Foundation staff Movement Disorder Specialist*
1:10 a.m. (20 min)	Post-test/evaluation/networking; certificate upon completion

In italics the professional figure taking care of the specific activity.

Based on these results, the *promotores de salud* could be employed to educate and could be involved in research on Hispanic patients with PD. Specifically, they could help design, explain, and disseminate information for research trials and, most importantly, build a local and permanent infrastructure to be used as a community resource for education and involvement in research.

### 4.2 Identification of needs and priorities with focus groups and surveys

In addition to the *promotores de salud*, the Parkinson's Foundation directly engages patients through the *Parkinson's Foundation Survey* and *semi-structured focus groups*. These instruments can provide better insights into the unique experiences, needs, and priorities of those affected by PD who identify as Hispanic. In particular, the *Parkinson's Foundation Survey* initiative invites people with PD, care partners, and medical professionals to share more about living with and treating PD. Surveys can be developed and launched to understand the needs, priorities, knowledge, and attitudes toward research in a specific community and to inform the development of a recruitment model for research. Surveys may be launched in a variety of ways to extend reach or define a target audience (i.e., Hispanic community), using the contacts established by the promotores as well as mailing lists of Hispanic organizations and those of the Parkinson's Foundation that include participants in PD GENEration (Cook et al., 2023), attendees of local, regional, and national Hispanic education as well as outreach events and constituents that identify as Hispanic that have contacted the Parkinson's Foundation helpline.

### 4.3 Gathering patients' feedback about their participation in research

Another important benefit to improving research participation is facilitating focus groups of Hispanic patients with PD participating in research studies to gather feedback about their experience. Work in this specific area is still in progress, and results are not yet available. Nevertheless, it is plausible that, in this respect, findings from the *Parkinson's Foundation Survey* and focus groups can provide a framework for improving recruitment models. Contributions to these models include the development of specific recruitment content for dissemination through many platforms, including the Parkinson's Foundation Spanish Facebook groups, targeted recruitment emails, personalized emails to coordinators of the Parkinson's Foundation Centers of Excellence, and scripts about the study for the Parkinson's Foundation Helpline Staff.

In summary, the training of *promotores de salud* can provide educational programs for Hispanic communities and can be used to monitor patients' feedback on their research experience. This, in turn, can give essential support for the recruitment and participation maintenance of Hispanic patients in research and clinical trials and, thus, more importantly, can lead to better care and access to care for Hispanic patients with PD.

## 5 Conclusions

The proposed approach will provide a greater understanding of the challenges associated with fostering Hispanic patient participation in clinical trials for PD and a working model for outreach, awareness, education, and recruitment for clinical PD studies in the Hispanic communities. Importantly, this approach could also be adapted for reaching other communities. Those in the Hispanic community suffering from PD are disproportionately impacted by the disease symptoms while at the same time being inadequately represented in clinical research. The development of this research engagement model in the Hispanic population will not only provide local infrastructure for outreach and clinical studies, such as the genetic studies proposed in the PD GENEration (Cook et al., 2023), but, most importantly, has the potential to become the means to educate patients and implement better clinical care for all communities and groups. Furthermore, this program can establish sustainable partnerships with community-based organizations to empower patients, promote research efforts, and disseminate knowledge about PD. Altogether, these strategies may become essential tools for improving Hispanic patients' health, enhancing their quality of life, and promoting initial steps toward universal PD care.

## Data Availability

The original contributions presented in the study are included in the article/supplementary material, further inquiries can be directed to the corresponding author.
